# 

**Published:** 1878-03

**Authors:** 


### CONTENTS:

ARTICLE I.—The Effect of Anæsthetics upon the Human System, as evidenced by Spectroscopic Observations. By Dr. S. Waterman, of New York.. 481
ARTICLE II.—What Anæsthetic Shall We Use? By Julian J. Chisholm, M. D.... 505
ARTICLE III—Substance of Lectures on Gold. By Prof. Hodgkin.............. 511

EDITORIAL, ETC.

Dr. Waterman on Spectroscopic Analysis.............. 518

Celluloid .............. 519

OBITUARY.

Professor George T. Barker. D. D. S.............. 520

John Ensor La Motte, D. D. S.............. 522

MONTHLY SUMMARY.

Unusual Action of Anæsthetics.............. 522

Chloral for Removing Warts.............. 523

The Liquefaction of Gases.............. 523

Supra-Orbital Neuralgia.............. 524

General Nervousness in the Male.............. 524

Death from Chloroform Averted by Nitrite of Amyl.............. 525

Subcutaneous Injections of Chloroform.............. 525

Can Twenty Grains of Chloral Kill ?.............. 526

The Odors of Persons.............. 526

Deaths from Anæsthetics in 1877.............. 526

Circassian Surgery.............. 527

Arsenic and Antimony in Vulcanized Rubber.............. 527

Chloroform Hallucination and Alleged Rape.............. 528

Iron Cement.............. 528

